# A Survey of Marine Natural Compounds and Their Derivatives with Anti-Cancer Activity Reported in 2011

**DOI:** 10.3390/molecules18043641

**Published:** 2013-03-25

**Authors:** Wamtinga Richard Sawadogo, Marc Schumacher, Marie-Hélène Teiten, Claudia Cerella, Mario Dicato, Marc Diederich

**Affiliations:** 1 Laboratory of Molecular and Cellular Biology of Cancer (LBMCC), Hôpital Kirchberg, 9, rue Edward Steichen, L-2540 Luxembourg, Luxembourg; 2 Institute of Health Sciences Research (IRSS), 03 BP 7192 Ouagadougou 03, Burkina Faso; 3 Department of Pharmacy, College of Pharmacy, Seoul National University, Seoul 151-742, Korea

**Keywords:** anticancer molecules, marine origin, synthetic derivatives, cancer

## Abstract

Cancer continues to be a major public health problem despite the efforts that have been made in the search for novel drugs and treatments. The current sources sought for the discovery of new molecules are plants, animals and minerals. During the past decade, the search for anticancer agents of marine origin to fight chemo-resistance has increased greatly. Each year, several novel anticancer molecules are isolated from marine organisms and represent a renewed hope for cancer therapy. The study of structure-function relationships has allowed synthesis of analogues with increased efficacy and less toxicity. In this report, we aim to review 42 compounds of marine origin and their derivatives that were published in 2011 as promising anticancer compounds.

## 1. Introduction

Despite significant advances in biomedical research during the last few decades, cancer remains a major cause of morbidity and mortality worldwide, with a likelihood of increasing prevalence [1,2]. The World Bank income groups estimated that the incidence of 12.7 million new cases in 2008 [3] will rise to 21.4 million by 2030 [2]. Therefore, it is imperative to find novel drugs and treatments to overcome this predicted situation. Because of the multiple side effects observed with chemotherapy, researchers are focusing more on drugs derived from natural products. From 1981 to 2010, natural products and their derivatives were the source of 41% of new drugs and 79.8% of all approved anticancer drugs [4]. Additionally, the percentage of drugs from natural products without derivatives was greatly increased from 20.8% in 2009 to 50% in 2010 [4]. Various molecules with anticancer properties were either isolated or derived from plants and terrestrial microorganisms, both of which have long-standing historical uses for the treatment of many diseases. This is not the case for marine organisms, where the bioactive compounds were first isolated in the early 1950s. The focus on the discovery of molecules of marine origin only began in the mid-20th century, with several hundreds of novel compounds being discovered every year [5,6]. There is actually use of marine organisms in traditional medicine around the World. Benkendorff reported in 2010 that mollusks in particular are listed in the Traditional Chinese *Materia Medica*. They appear in traditional South African invertebrate medicine markets and are also used in Indian and Pacific Island remedies [7]. A significant number of anticancer molecules of marine origin have been isolated, but only a few of them are available on the market. According to the records of the Food and Drug Administration (FDA) and those of the European Agency for the Evaluation of Medicinal Products (EMA), from 1940 to 2010, 113 drugs (including natural compounds and their derivatives) were approved in cancer treatment, but only three of them (2.65%) are of marine origin. These compounds were introduced between 1993 and 2010 with the trade names of Starsaid^®^ (cytarabine, introduced in 1993), Yondelis^®^ (trabectedin, 2007) and Halaven^®^ (eribulin, 2010) [4]. It was estimated that 118 marine anticancer molecules are presently in pre-clinical trials whereas 22 other molecules are undergoing clinical trials [8]. These data highlight the nascent stages of marine anticancer drug development. The marine environment contains a great diversity of organisms exhibiting a variety of molecules with unique structural features not found in terrestrial natural products. Because of the great interest in marine bioactive compounds, researchers isolated hundreds of compounds each year and evaluated them for their anticancer properties. In 2011, our team reported a selection of 13 groups of marine natural anticancer compounds and their associated analogues published in 2010 [9]. This review is a continuation of that report with the aim to review a selection of 42 marine compounds and their derivatives reported in 2011 as promising candidates for anticancer drug development. Among them, about 4.7% were already mentioned in our previous report; 45.2% are novel structures and 50% are described as anticancer agents for the first time in 2011. Their anticancer properties will be discussed and their structure–function relationships investigated.

## 2. Promising Marine Anticancer Molecules Reported in 2011

### 2.1. Alkaloids

#### 2.1.1. Agelasine analogs 2F and 2G

**Figure 1:**
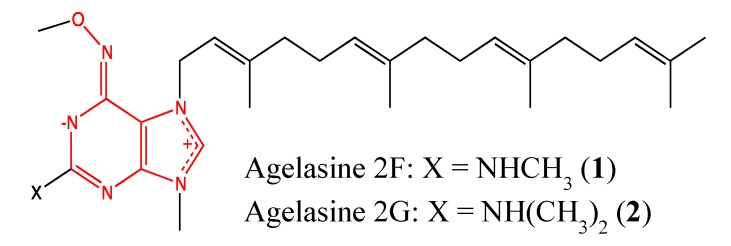
Chemical structure of Agelasine 2F and 2G.

Agelasines are 7,9-dialkylpurinium salts found in marine sponges and were first reported in 1984 by Nakamura *et al.* [10]. The primary chemical structure was modified by the introduction of either a dimethylamine group or its extended form at the purine 2-position, which resulted in the two cytotoxic analogs 2F and 2G (**1**, **2**, Figure 1). These compounds showed high cytotoxicity against a panel of cancer cells (U937-GTB, RPMI8226, CEM, ACHN), with IC_50_ values ranging from 0.55 to 4.2 μM [11]. This chemical modification resulted in a two- and three-fold increase in the toxicity of these analogs against U-937 and ACHN cells, respectively. Thus, dimethylamine and its extended groups are relevant pharmacophores in the anticancer properties of agelasine analogs. These compounds need to be further investigated to elucidate their mechanisms of cytotoxicity and determine their toxicity to healthy cells because the parent compound showed significant cytotoxicity against both Vero and MRC-5 cells, with IC_50_ values of 2 and 1.4 μM, respectively.

For all chemical structures (compounds **1–42**), the moieties responsible for their cytotoxicity are highlighted, as well as their ability to either enhance or reduce this effect. Moieties in red color are responsible for the cytotoxicity, those in blue enhance its effectiveness and those in green reduce its effectiveness. Each chemical structure was drawn using the software ChemBioDraw Ultra, version 12.0.3.1216 (Cambridge Soft Corporation, Cambridge, MA, USA) and is in conformity with the original structures published in the literature.

#### 2.1.2. Fascaplysin

**Figure 2:**
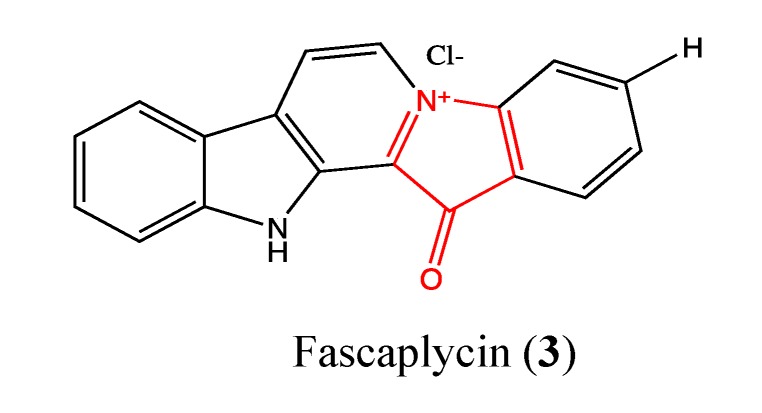
Chemical structure of Fascaplycin.

Fascaplysin (**3**, Figure 2) is a red pigment that was isolated from the marine sponge *Fascaplysinopsis bergquist* sp. in 1988 [12,13,14]. Previous studies showed the effects of this molecule on pathways and proteins that can inhibit cancer such as cell cycle arrest, cyclin-dependent kinase (CDK4)-specific inhibition, inhibition of vascular endothelial growth factor (VEGF) expression, anti-angiogenesis activity [15,16], DNA binding properties [17] and apoptotic effects through the activation of caspase-3, -8 and -9, bid truncation, release of cytochrome c and down-regulation of Bcl-2 [18]. Fascaplysin is cytotoxic toward a panel of 60 cancer cell lines [14]. In 2011, Yan *et al*., performed an *in vivo* study in a sarcoma mice model to confirm the effects of this molecule [12]. Their findings indicate that fascaplysin at tolerated doses in mice can inhibit the growth of S180 cell implanted tumors through apoptosis, anti-angiogenesis or cell cycle arrest, which confirms the *in vitro* results reported by many studies. Recently, Shafiq and co-workers confirmed the specific effect of this compound on CDK4, which is known to play a key role in the cell cycle and is a popular target for anticancer drugs [19].

#### 2.1.3. Makaluvamine Analog FBA-TPQ

**Figure 3:**
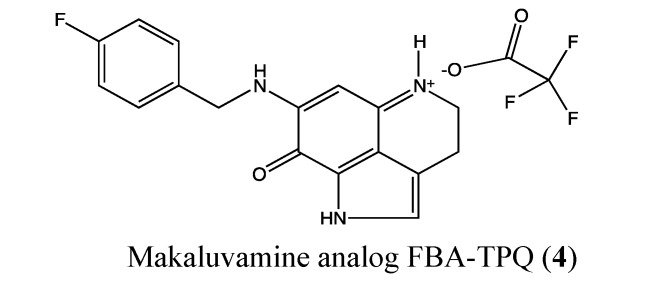
Chemical structure of Makaluvamine analog FBA-TPQ.

FBA-TPQ (**4**, Figure 3) is a synthetic analog of the natural makaluvamines, which are marine pyrroloiminoquinone alkaloids that have been isolated from sponges of the genera *Zyzzya*. Previous studies showed that FBA-TPQ is able to inhibit tumor growth by activating tumor suppressors and DNA damage response as well as inhibiting oncogene expression [20,21]. In 2011, Chen *et al.* reported the possible use of this compound in ovarian cancer therapy. It significantly inhibited A2780 and OVCAR-3 ovarian cancer cells, with IC_50_ values of 1.78 and 0.98 μM, respectively [22]. The cytotoxic effect is reduced against non-tumorigenic IOSE-144 cells, with an IC_50_ value of 10 μM. Moreover, FBA-TPQ induces apoptosis and cell cycle arrest at 2.5 μM in OVCAR-3 cells. *In vivo*, it significantly inhibited OVACAR-3 xenograft tumor growth [22]. Considering these preliminary data, FBA-TPQ could be a potential lead for the development of ovarian anticancer drugs.

#### 2.1.4. Zalypsis (PM00104)

**Figure 4:**
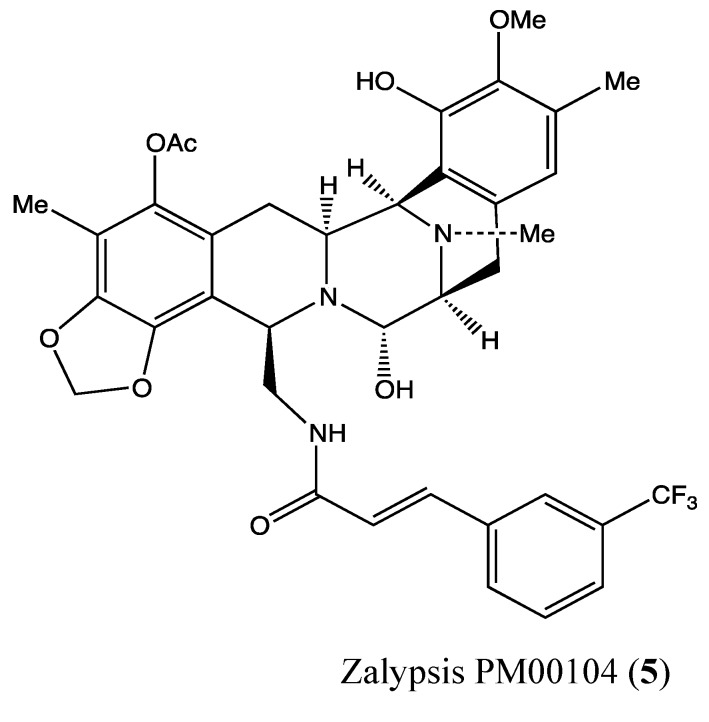
Chemical structure of Zalypsis PM00104.

Zalypsis (**5**, Figure 4) is a novel synthetic tetrahydroisoquinoline alkaloid of marine origin that demonstrated potent *in vitro* and *in vivo* inhibitory effects against both human solid and hematologic neoplasms [23,24]. In 2011, Colado *et al*. reported that Zalypsis exerts potent antileukemic activity in four cell lines (HEL, HL-60, MV4-11, KG1), with IC_50_ values below 1 nM after 24 h of treatment [25]. When combined with conventional drugs such as cytarabine, fludarabine and daunorubicin, Zalypsis increased the effects of these drugs against HEL and HL-60 cells as well as acute myeloid leukemia (AML). At a dose of 10 nM, it induced apoptosis in the two tested cell lines by both the intrinsic and extrinsic pathways through activation of caspases 3, 8, 7 and 9 and PARP cleavage between 12 to 24 h after treatment. This apoptosis was found to be the result of profound deregulation of several genes involved in cell survival (especially BRCA1) and in the recognition of double-stranded DNA breaks, with RAD51 and RAD54 specifically implicated in the repair of double-stranded DNA breaks [25]. In a recent phase I clinical trial, Zalypsis showed good tolerance and preliminary evidence of its antitumor activities against several solid tumors, such as cervical carcinoma, colorectal adenocarcinoma, lachrymal adenoid carcinoma and bladder carcinoma [26,27,28]. This positive risk-benefit ratio has supported its continued evaluation in three ongoing phase II clinical trials.

### 2.2. Anthraquinones

#### 2.2.1. Doxorubicin Analogue 1403P-3

**Figure 5:**
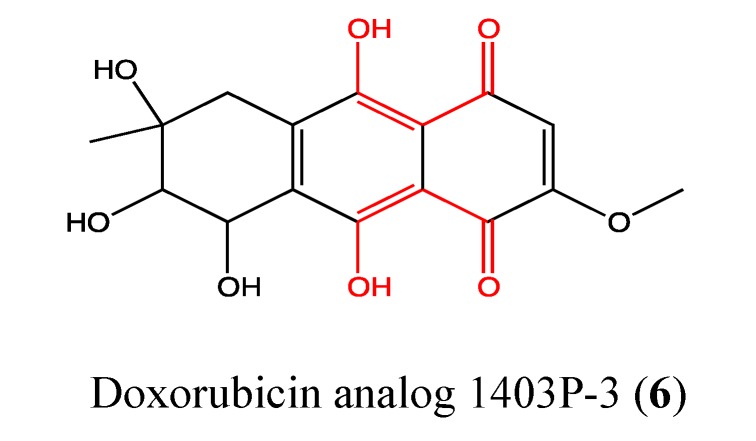
Chemical structure of Doxorubicin analog 1403P-3.

Doxorubicin analogue 1403P-3 (**6**, Figure 5) was isolated from the mangrove endophytic fungus No. 1403 from the South China Sea and reported for the first time in 2000 [29]. 1403P-3 is an anthracenedione derivative with potent anticancer activities. In 2007, Zhang *et al*. reported a significant cytotoxic effect of this compound on both KB and MDR-KBv200, with an IC_50_ value of approximately 19 μM [30]. This toxicity acts through apoptosis by the loss of the mitochondrial membrane potential, release of cytochrome *c*, cleavage of Bid and activation of caspases 2, 3, 7, 8 and 9. DNA fragmentation and PARP cleavage were also observed. In 2011, Yuan *et al*. reported a high cytotoxic effect of 1403P-3 on MCF-7 and MDA-MB-435 cells, with IC_50_ values of 9.5 and 7.6 μM, respectively [31]. These cell lines are more sensitive to 1403P-3 than KB cells [30,31]. As expected, the toxicity of this compound on MCF-7 and MDA-MB-435 cells is triggered through apoptosis by the activation of caspases 8 and 9 and cleavage of PARP. Akt activation was blocked in the cells treated with 1403P-3, and the level of ROS was significantly decreased in these cells. Akt is a well-characterized apoptosis-related signaling molecule involved in sustaining survival against apoptosis in cancer cells [31,32]. Upregulation of Akt in cancer cells is a common event, and the activation of the PI3K/Akt pathway leads to chemo-resistance [33,34,35]. The fact that 1403P-3 can block Akt activation by reducing the amount of phosphorylation is an important attribute for cancer therapy [36]. Regarding the chemical structure of 1403P-3, the presence of phenolic hydroxyl groups in the primary backbone of the molecule is responsible for its antioxidant activity [30], as observed by the decrease in the amount of ROS in KB cells, whereas the quinone moiety has been implicated in its cytotoxic effects. 

#### 2.2.2. Bostrycin

**Figure 6:**
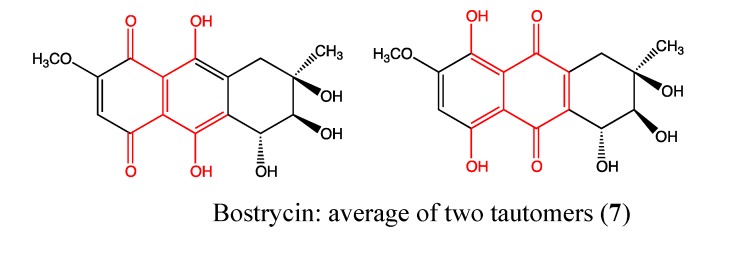
Chemical structure of Bostrycin.

Bostrycin (**7**, Figure 6) is a novel anthracene analog (hydroxymethoxytetrahydro-5-methylanthracenedione) that was isolated from marine fungi in the South China Sea and reported in 2008 by Lin and coworkers [37]. It was previously shown to be a potent inhibitor of cell growth and promoter of apoptosis in prostate and gastric cancer cells. In 2011, Chen *et al*. demonstrated its significant inhibitory effect on A549 lung cancer cell proliferation in a dose and time dependent manner, with an IC_50_ value of less than 10 μM after 72 h of treatment [37]. The chemical structure of this compound contains a quinone moiety that is responsible for its cytotoxicity. Additionally, the phenolic hydroxyl groups enhance its effect through the reduction of ROS levels [30]. This compound causes cell cycle arrest in the G0/G1 phase and induces apoptosis through up-regulation of microRNA-638 and -923 as well as down-regulation of PI3K/AKT pathway proteins [37]. These findings suggest that bostrycin may lead to the development of a PI3K/AKT targeting drug in the treatment of lung cancer.

### 2.3. Benzothiazoles

**Figure 7:**
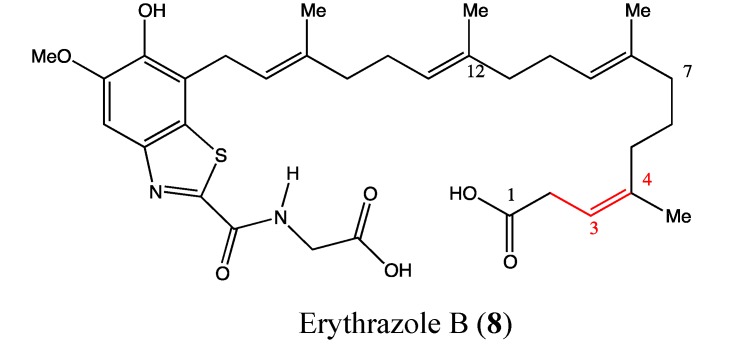
Chemical structure of Erythrazole B.

Erythrazole B (**8**, Figure 7) is a benzothiazole that was isolated as a light yellow crystal from the marine bacterium *Erythrobacter sp*. It contains a tetrasubstituted benzothiazole, an appended diterpene side and a glucine unit. Erythrazole B was found to be highly toxic against H1395, H2122 and HCC366 cell lines, with IC_50_ values of 1.5, 2.5 and 6.8 μM, respectively [38]. The double bond between C3-C4 appears to be crucial for the cytotoxicity of this compound because its removal increases the IC_50_ value to 20 μM. As benzothiazoles are relatively rare as natural products, this compound requires further investigation to determine its possible uses in cancer therapy.

### 2.4. Macrolides

#### 2.4.1. Apratoxin Analog S4-1e

**Figure 8:**
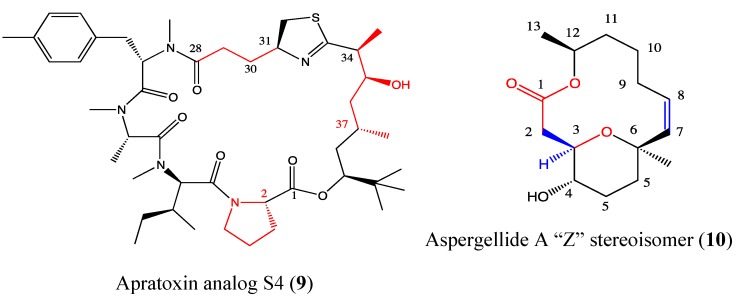
Chemical structure of Apratoxin analog S4 and Aspergellide A.

Apratoxins are produced by marine cyanobacteria and are potent toxins that are known to prevent cotranslational translocation early in the secretory pathway, which can be exploited in cancer therapy [39]. The aberrant pro-growth signaling in cancer cells is due to increased ligand binding to their receptor tyrosine kinases (RTKs). These enzymes are implicated in the regulation of multiple cellular processes that contribute to tumor development and progression [40]. Thus, the inhibition of the binding of these enzymes to their ligands is a target in cancer therapy. This interaction can be blocked by targeting both ligands and receptors. This “one-two punch” emerges as an effective alternative to combination therapy, especially in cancers (e.g., colorectal cancer) where secreted growth factors play a major role. Chen *et al*. reported the use of the apratoxin analog S4-1e (**9**, Figure 8) in this one-two punch therapy [39]. This molecule was obtained through hybridization of parent compounds apratoxin A and E. S4-1e is five times more cytotoxic than apratoxin A and 161 times more than apratoxin E. This compound also inhibits VEGF-A secretion, an angiogenic drug target [41], 5-fold more than apratoxin A and 29 fold more than apratoxin E. S4-1e also exhibits a better tolerance *in vivo* compared to apratoxin A. This compound has emerged as the first relevant candidate of the apratoxin family because of its selectivity and efficacy in colorectal cancer [39]. Several studies of the structure-function relationship of the apratoxin family showed that modification on the configuration at C-2 and C-37 is crucial for potent cytotoxicity whereas modifications at the C33-C43 and C28-C31 chains can reduce the effectiveness of this compound [42,43,44]. The complex chemical structure of S4-1e is one example of many pharmacophores that has a justifiable one-two punch in cancer therapy.

#### 2.4.2. Aspergillide A Analog

Aspergillides are macrolides that were isolated from a strain of marine-derived fungus *Aspergeillus ostianus* and have unusual features. Aspergillide A (**10**, Figure 8) was described to exert moderate toxicity against murine lymphoma (L1210) cells [45]. A chemical modification of aspergillide A led to its Z stereoisomer that induces high toxicity against a panel of cancer cells (HL-60, MDA-MB-231, HT-1080, HT-29, U2OS) [46]. Its toxicity was found to be 34 and 45 times higher than the parent compound in breast cancer and leukemia cell lines, respectively. The highest cytotoxicity of the aspergillide A Z stereoisomer was observed in leukemia cells, with an IC_50_ value of 1.8 μg/mL, which is comparable to that of the clinical drug fludarabine. The configuration of the C-2 and the “H” in C-3 as well as the double bond in C7-C8 enhance the cytotoxicity of “Z”-aspergillide A. The selectivity of its cytotoxicity need to be proved on PBMC cells before considering this compound as lead to the development of anti-leukemia drugs in preclinical and clinical trials.

#### 2.4.3. Chromomycin SA_2_

**Figure 9:**
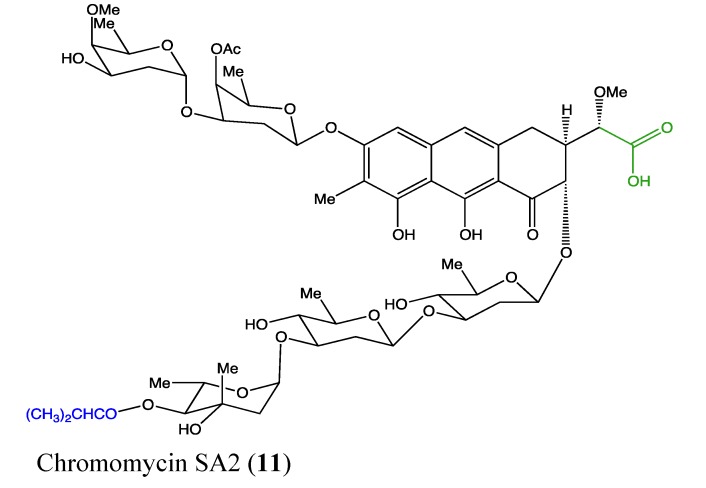
Chemical structure of Chromomycin SA2.

Chromomycin SA2 (**11**, Figure 9) is a glycosylated polyketide that was recently isolated from the marine-derived *Streptomyces sp*. This novel molecule exhibits high cytotoxicity against two non-small cell lung cancer A549 and HCC44 cells, with IC_50_ values of 1.5 and 0.78 μM, respectively [47]. The carboxylic acid moiety in its chemical structure was found to be deleterious to the cytotoxicity of this compound. In general, chromomycin family members, including chromomycin A2, A3 and SA, are effective against many types of human cancers [47,48]. The recently discovered chromomycin SA2 requires further investigation to better understand its cytotoxic effects on cancer cells and its possible use in therapies.

#### 2.4.4. Lobophorin C and D

**Figure 10:**
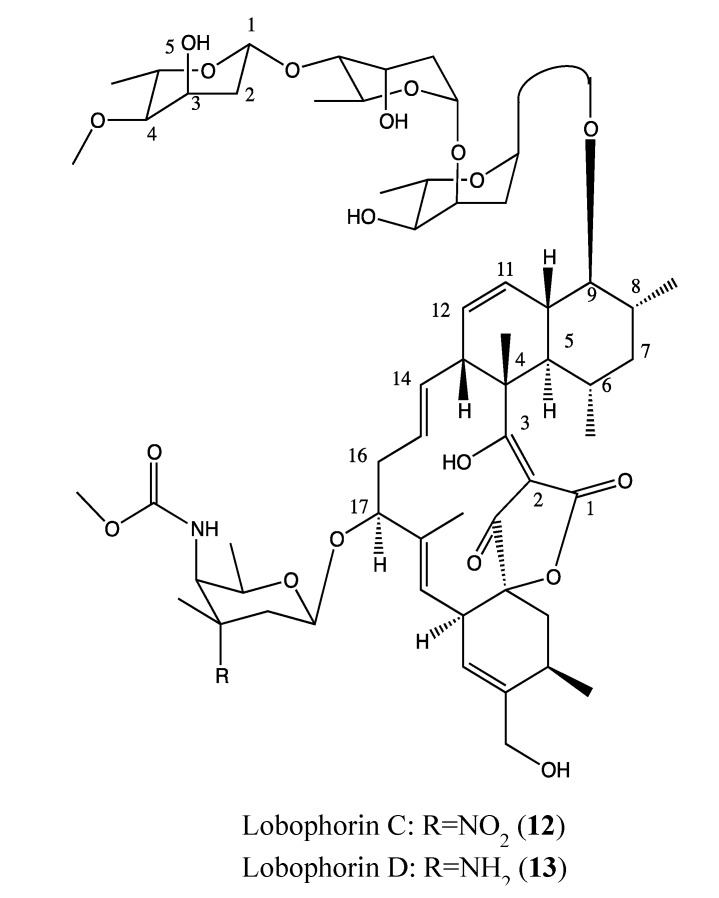
Chemical structure of Lobophorin C and D.

Lobophorin C and D (**12**, **13**, Figure 10) are novel kijanimicin derivatives that were isolated from the marine sponge-associated actinomycetal strain AZS17. These compounds have a similar chemical structure except for one difference in the radical of the sugar moiety at position 17. Interestingly, they display a selective cytotoxicity against breast cancer cells (MDA-MB-435) and human liver cancer cells (Bel-7402). Lobophorin C exhibits strong toxicity against 7402 hepatoma cells (IC_50_ of 0.6 μg/mL) but is inactive in MDA-MB-435 cells, whereas lobophorin D exerts its toxicity toward MDA-MB-435 cells (IC_50_ of 7.5 μM) but is inactive against Bel-7402 [49]. This biological difference is linked to their difference in the radical of a sugar moiety in position 17 of each compound. These two compounds require further investigation as a combinatory regimen to determine if there is a possible synergetic effect. 

**Figure 11:**
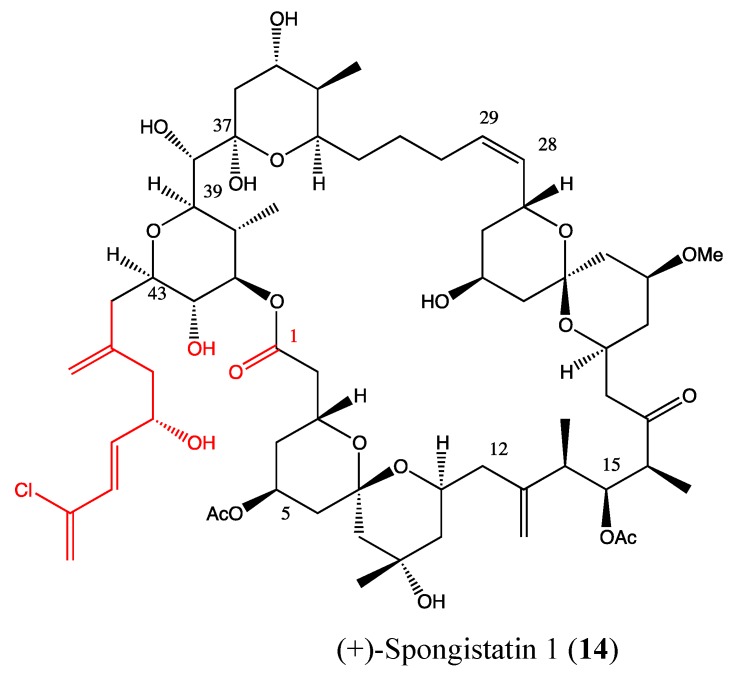
Chemical structure of (+)-Spongistatin 1.

2.4.5. (+)-Spongistatin 1

(+)-Spongistatin (**14**, Figure 11) is a natural marine compound with a high molecular weight and complex chemical structure [50,51]. It was recognized as the most cytotoxic compound within the spongistatin family against a wide variety of cancer cells, with a minimal effect on quiescent human fibroblasts [52,53]. Several studies revealed its effects on inhibiting microtubule activity and metastasis as well as promoting apoptosis [54,55,56]. In 2001, Xu *et al*., reported a confirmation of its extreme cytotoxicity against a panel of 13 cancer cells at sub-nanomolar levels, with IC_50_ values ranging between 0.037 and 0.5 nM [57]. Interestingly, it was found to be 10,000-fold less toxic to IMR-90 quiescent fibroblasts. This compound caused mitotic arrest in human prostate cancer (DU145) cells and a significant inhibitory effect on tumor growth *in vivo* in a human melanoma xenograft model. The various properties of this molecule are likely related to the complexity of its chemical structure, especially the presence of the full C44-C51 side-chain, which was found to be crucial in its bioactivity [53].

### 2.5. Peptides

#### 2.5.1. Acetylapoaranotin

**Figure 12:**
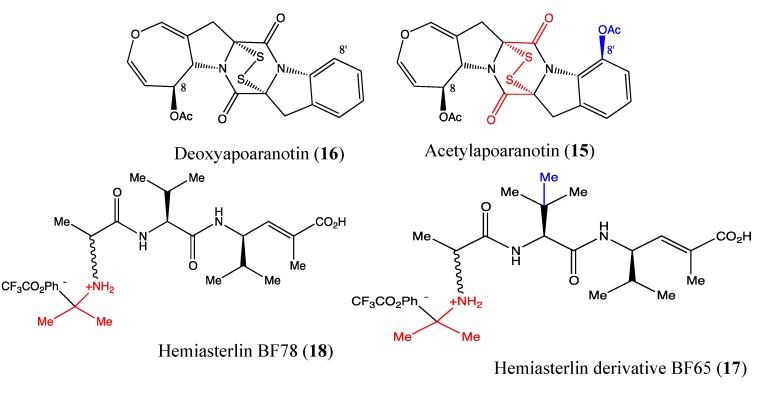
Chemical structure of Apoaranotin analogs and Hemiasterlin derivatives.

Acetylapoaranotin (**15**, Figure 12) is a diketopiperazine disulfide that was isolated from marine *Aspergillus sp*. KMD 901. In 2011, Choi and co-workers reported the benefit of this compound for cancer therapy [58]. Indeed, acetylapoaranotin is cytotoxic to HCT116, AGS, A549 and MCF-7 cells at IC_50_ values of 13.8, 12, 2 and 10 μM, respectively. Specifically, it induces apoptosis in human colon cancer cells (HCT116) as demonstrated by DNA fragmentation, annexin-V/PI staining and PARP, and caspase-3, -8, -9, Bcl-2, Bcl-xL and Bax cleavage. This compound also significantly inhibits tumor growth in mice *in vivo* [58]. The chemical structure of acetylapoaranotin contains disulfide bridges, which are able to generate reactive oxygen species and are responsible for the molecule’s cytotoxicity [59]. The O-acetyl group in 8’ position appears to enhance the cytotoxicity of this molecule because deoxyapoaranotin (**16**, Figure 12), its analog without the O-acetyl group is less toxic. Nevertheless, further studies are necessary to better understand the mechanism of action of this compound.

#### 2.5.2. Hemiasterlin Derivatives BF65 and BF78

Hemiasterlin compounds are cytotoxic tri-peptides isolated from marine sponges. They are known tubulin inhibitors, and some of them, including hemiasterlin HTI-286 and E7974, are in early-phase clinical trials [60,61,62]. The novel hemiasterlin derivatives BF65 (**17**, Figure 12) and BF78 (**18**, Figure 12) showed high toxicity at nanomolar levels toward a panel of cancer cell lines, including A549, H1299, UCI-101, MDA-MB231, SNU-423, MiaPaca2 and HCT116 [63]. These two compounds have a similar chemical backbone with a small difference due to an additional methyl group in BF65, which enhances its cytotoxicity. This compound induces cell cycle arrest at G2/M phase; this finding confirms this compound’s inhibitory effects on tubulin. The two compounds induce apoptosis in the same manner as vincristine, which was used as a positive control. Bcl-2 phosphorylation and caspase-3 activation were observed after 16 hours. Moreover, these compounds were able to block tubulin polymerization at a low dose (5 μM). Finally, the analog BF65, which is the more active compound than BF78, suppresses tumor growth in the mouse xenograft model. The beneficial effects of these compounds are enhanced by combinatorial treatment with stilbene 5c [63]; this synergistic effect could be highly useful in combinatory therapies. 

#### 2.5.3. Jasplakinolide V

**Figure 13:**
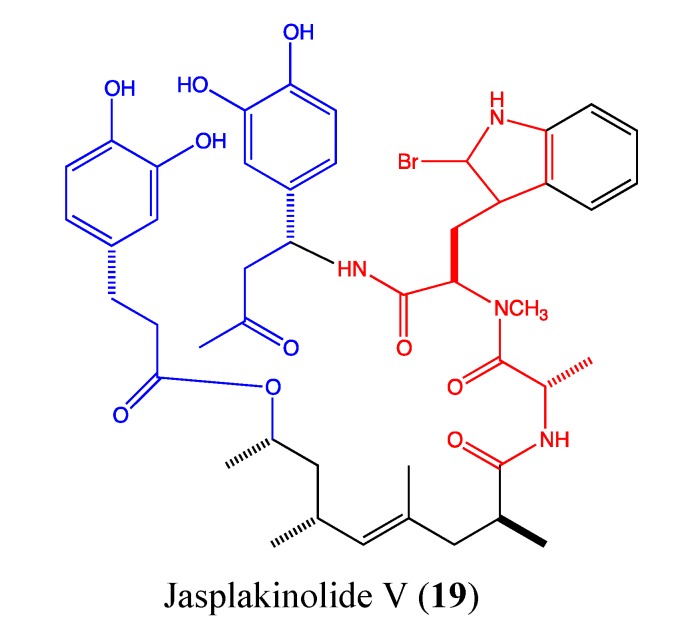
Chemical structure of Jasplakinolide V.

Jasplakinolide V (**19**, Figure 13) is one of the cyclodepsipeptide jasplakinolide congeners isolated from the marine sponge *Jaspis splendens*. This novel compound exerts a high cytotoxicity at the nanomolar level against a panel of cancer cells including HCT-116 (GI_50_ of 0.07 μM), MDA-MB-231 (IC_50_ of 0.09 μM), IGROV-1 (IC_50_ of 0.03 μM), A498 (IC_50_ of 0.01 μM), LOX-IMVI (IC_50_ of 0.007 μM), U25-1 (IC_50_ of 0.04 μM), NCI-H522 (IC_50_ of 0.06 μM) and DU-145 (IC_50_ of 0.08 μM). Jasplakinolide V induces total loss of the microfilament network at 0.5 μM in HCT-116 cells [64]. This activity is due to the geometry of the Ala-N-Me-2BrTrp-β-Tyr segment in its chemical skeleton, which allows for binding to the actin site. The key components of this active binding involve the Trp ring system of the molecule interacting with the aromatic amino acids of filamentous actin and the contact of the Tyr-OH functional group with an actin threonine residue [65]. This cytotoxic compound with its interesting stabilization property on filamentous actin is a promising anticancer agent and requires more investigation to determine its mechanism of action.

#### 2.5.4. Lagunamide C

**Figure 14:**
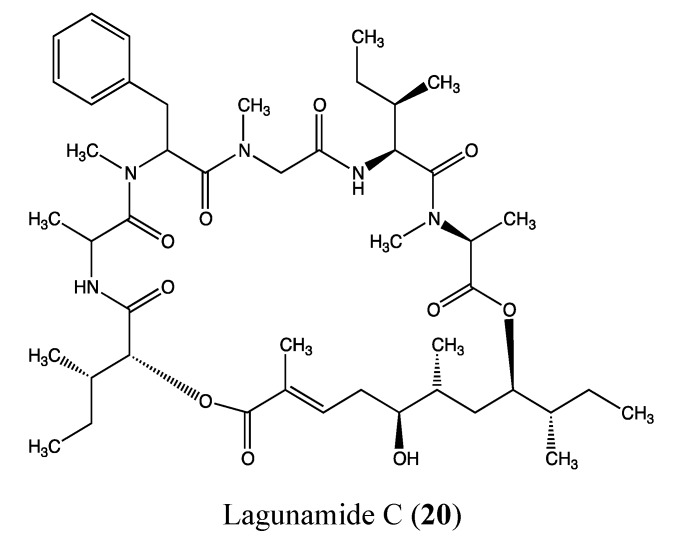
Chemical structure of Lagunamide C.

Lagunamide C (**20**, Figure 14) is a cytotoxic cyclodepsipeptide that was isolated from *Lyngbya majuscula*, a marine cyanobacterium that produces structurally diverse bioactive compounds [66]. This compound exhibits potent cytotoxic effects at the nanomolar level toward a panel of cancer cells, including P388 (IC_50_ of 24.4 nM), A549 (IC_50_ of 2.4 nM), PC3 (IC_50_ of 2.6 nM), HCT8 (IC_50_ of 2.1 nM) and SK-OV (IC_50_ of 4.5 nM) [67]. Lagunamide C is also potent against *Plasmodium falciparum*, with an IC_50_ value of 0.29 μM, confirming the fact that antimalarial compounds can also possess anticancer activity [68]. The particular features of the chemical structure of this compound and its high toxicity against several cancer cell lines are a basis for further biological investigations to determine the possible applications of lagunamide C in cancer therapies.

#### 2.5.5. Largazole

**Figure 15:**
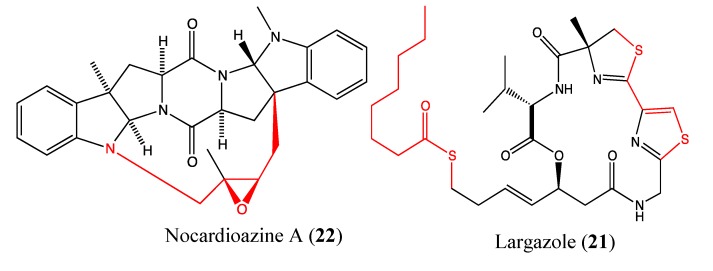
Chemical structure of Largazole and Nocardioazine A.

Largazole (**21**, Figure 15) is a macrocyclic depsipeptide that was isolated from the marine cyanobacterium *Symploca* sp. Its core structure is similar to the chemical structure of romidepsin, which is a natural depsipeptide formulated as Istodax that was recently approved for the treatment of cutaneous T-cell lymphoma [69]. Largazole is a highly potent histone deacetylase (HDAC) inhibitor at picomolar concentrations [70,71,72,73]. HDAC inhibitors block the proliferation of tumor cells by inducing cell differentiation, cell cycle arrest, and/or apoptosis [74]. As expected, largazole inhibited the growth of HCT-116 and MCF-7 cells, with IC_50_ values of 10 and 5 nM, respectively [72,73].

#### 2.5.6. Nocardioazine A

Nocardioazine A (**22**, Figure 15) is a novel marine diketopiperazine that was isolated from the bacterium *Nocardiopsis* sp. (CMB-M0232). This molecule does not affect the viability of cancer cells but instead inhibits the expression of P-gp at 20 μM with a similar efficacy as 10 μM of the reference drug verapamil. This property appears to be linked to the novel bridged scaffold in its chemical structure. Additionally, it is able to reverse doxorubicin resistance in SW620Ad300n colon cancer cells [75]. Thus, nocardioazine A appears to be a promising noncytotoxic P-gp inhibitor that could be used in a combinatory therapy to overcome the MDR phenomena.

#### 2.5.7. Pardaxin

Pardaxin is a polypeptide with 33 amino acids that was isolated from the secretions of the Red Sea Moses sole (a small fish). Pardaxin is known to possess antibacterial, antiviral and neurotoxic activities. In 2011, Hsu *et al.* and Huang *et al.* demonstrated its cytotoxic effect against Hela and HT-1080 cells, respectively [76,77]. Its toxicity is low against normal fibroblast WS-1 cells at 15 μg/mL after 24 h of exposure. Pardaxin induces apoptosis in HT-1080 cells via depolarization of the mitochondrial membrane potential (MMP) and release of cytochrome c. It also increases ROS production and triggers the activation of caspases 3 and 7, which lead to mitochondrial-dependent apoptosis [76].

### 2.6. Sphingolipids

**Figure 16:**
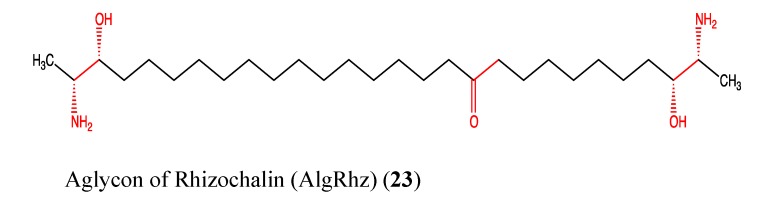
Chemical structure of Aglycon of Rhizochalin.

Rhizochalin is a two-headed sphingolipid-like molecule that was isolated from the marine sponge *Rhizochalina incrustata* [78,79]. Several analogs of this compound were either isolated or synthetized since its discovery in 1989 [78]. One of them is its aglycon, which is rhizochalin without its sugar moiety (**23**, Figure 16). Since 2009, the aglycon of rhizochalin (AglRhz) was found to be cytotoxic to many cancer cell lines, such as uterine, cervical and colon cancers as well as leukemia [80,81]. Its mechanism of action was elucidated in 2011 through the work of Khanal *et al.*, who demonstrated that AglRhz induces AMPK, caspase-3 and PARP activation as well as DNA fragmentation in HT-29 cells [79]. All of these effects result in apoptosis and suppression of the tumorigenicity of HT-29 cells. Moreover, AglRhz inhibits insulin-like growth factor (IGF)-1-induced AP-1 activity and cellular transformation in JB6 Cl 41 cells [79]. It is known that AMPK has a role in metabolism and cell growth regulation and thus has become a target in cancer therapy. AP-1 activation, through the elevation of circulating IGF-I levels, plays a pivotal role in tumorigenesis and mediates an anti-apoptotic response to both chemotherapy and radiotherapy in cancer cells [82,83]. AMPK phosphorylation by AglRhz results in the inhibition of AP-1 via a cascade of biochemical reactions. This finding was confirmed by the significant inhibition of IGF-I-induced neoplastic cell transformation of JB6 Cl 41 cells [79]. The chemical structure of AglRhz shows the presence of a ketone moiety in the middle of the backbone and amine and hydroxyl moieties at each head (**23**). Theses moieties are responsible for the pharmacological properties of AglRhz. Additionally, aglycon is less polar than the parent compound and can easily cross the lipid bilayer of cell membranes.

### 2.7. Steroids

**Figure 17:**
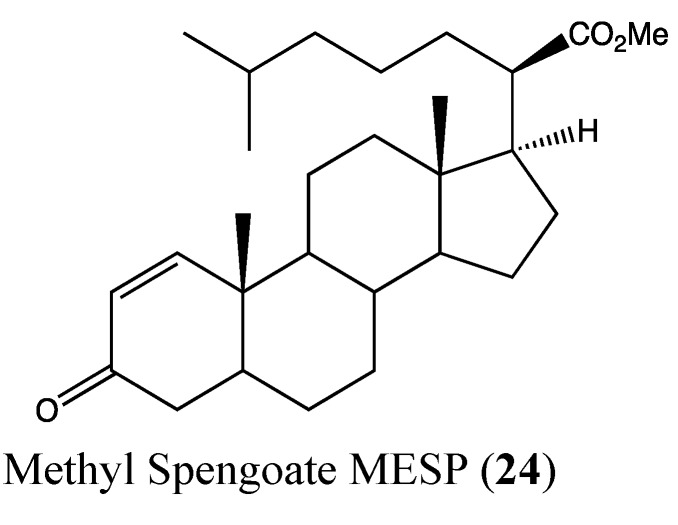
Chemical structure of Methyl Spongoate.

Methyl spongoate *(MESP)* (**24**, Figure 17) is a novel marine steroid that was isolated from the Sanya soft coral *Spongodes* sp., with a unique chemical feature due to the presence of a 21-oic-acid methyl ester moiety with 20R configuration [84]. MESP displays potent toxicity against six hepatocellular carcinoma (HCC) cell lines, with IC_50_ values ranging from 1.7 to 9 μM. It is known that advanced hepatocellular carcinomas are generally resistant to anticancer drugs because of the multidrug resistant (MDR) phenomena such as P-gp overexpression [85,86]. Interestingly, MESP is cytotoxic toward the MDR-positive tumor cells, including MCF-7/ADR and KB/VCR, without modulating the function of drug transporters. Additionally, this molecule does not affect steroid hormone-dependent cancer signaling, cell cycle signaling, microtubule function or the activities of topoisomerase and tyrosine kinase. The cytotoxicity mechanism of MESP was observed to act through the apoptotic pathway by inhibiting STAT3 and activating caspase-3, -8 and -9. The transcription factor STAT3 is known to play critical roles in human cancer formation and progression by regulating cell proliferation and antagonizing apoptosis [87,88]. MESP is able to regulate the balance between the anti-apoptotic and pro-apoptotic signals in cancer cells by relieving the former (effect on XIAP) and enhancing the latter (effect on Bax) [84]. All of these findings suggest that MESP has a singular cytotoxic mechanism against HCC cells. 

### 2.8. Tannins

**Figure 18:**
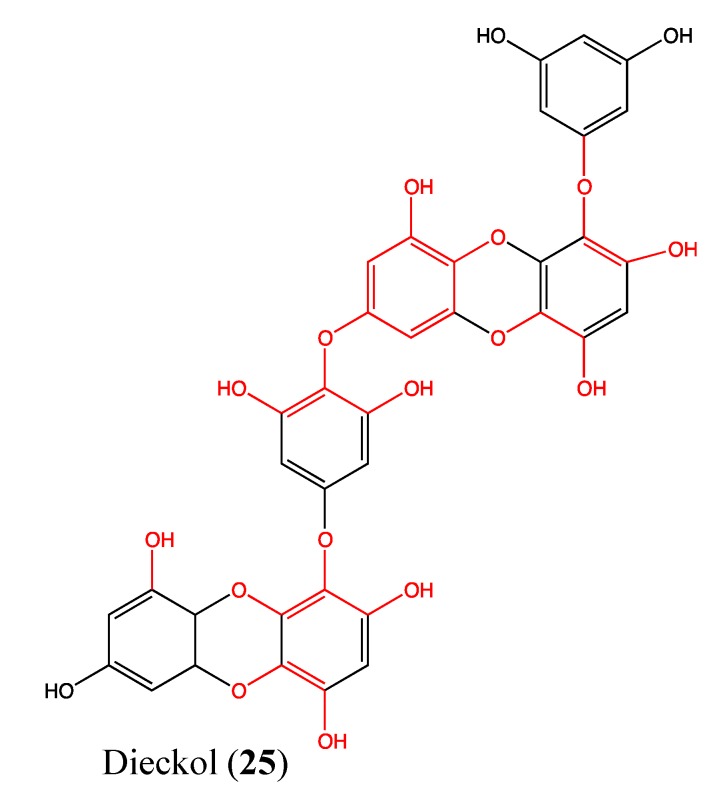
Chemical structure of Dieckol.

Dieckol (**25**, Figure 18) is a phloroglucinol (family of tannins) that was isolated from the marine brown alga *Ecklonia cava*. This compound is non-toxic to human fibrosarcoma cells (HT1080) below doses of 200 μM after 48 h of treatment. It inhibits the expression of the matrix metalloproteinase family (MMP-2 and MMP-9) in a dose-dependent manner and suppresses HT1080 cell invasion. The cytomorphology changes of these cells in a 3D culture system is also suppressed at non-toxic doses of dieckol (10 to 100 μM) [89]. It is known that both MMP-2 and MMP-9 play a crucial role in the establishment of metastasis; thus, these inhibitors have an obvious benefit in cancer therapy [90,91,92]. Recently, Park and Jeon demonstrated that dieckol inhibits HT1080 cell migration and invasion via ROS scavenging [93]. Additionally, dieckol inhibits the NF-κB pathway without effecting either the activator protein-1 (AP-1) pathway or tissue inhibitor of metalloproteinases (TIMPs) [89]. This type of mechanism of action could be due to the structural features that are specific to dieckol [94]. It contains 11 hydroxyl groups, which play an important role in its pharmacological effects. The three ether linkages in the skeleton provide more free anions for the attraction of receptors implicated in the therapeutic effects to cancer [89]. 

### 2.9. Terpenes/Terpenoids

#### 2.9.1. Astaxanthin

**Figure 19:**
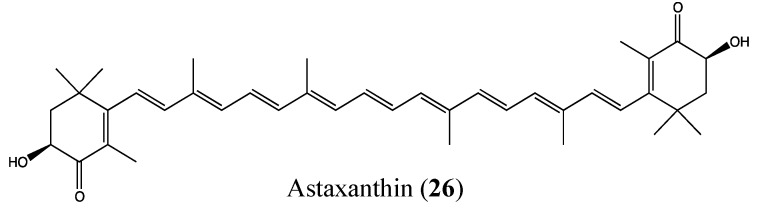
Chemical structure of Astaxanthin.

Astaxanthin (**26**, Figure 19) is a red-orange colored carotenoid from marine origin that is already well known for its powerful antioxidant, anti-inflammatory and anticancer properties [95,96,97,98,99,100]. In 2011, Yasui *et al*. demonstrated the *in vivo* inhibition effect of this compound on the expression of tumor necrosis factor (TNFα), nuclear factor kappa B (NF-κB) and interleukin 1β (IL-1β), as well as some inflammatory cytokines that play an important role in tumor promotion [101]. Astaxanthin (AX) inhibited cell proliferation and induced apoptosis in colon cancer [101]. When AX is given to mice at 200 ppm in their diet, the protein expression of NF-κB and the mRNA expression levels of IL-1β, IL-6 and cyclo-oxygenase-2 (COX-2) were decreased. It is known that NF-κB regulates the expression of several genes, such as COX-2, the matrix metalloproteinase MMP-9, iNOS, TNFα, IL-8, and other anti-apoptotic proteins, involved in tumor initiation, promotion and metastasis [102,103].

#### 2.9.2. Culobophylin A

**Figure 20:**
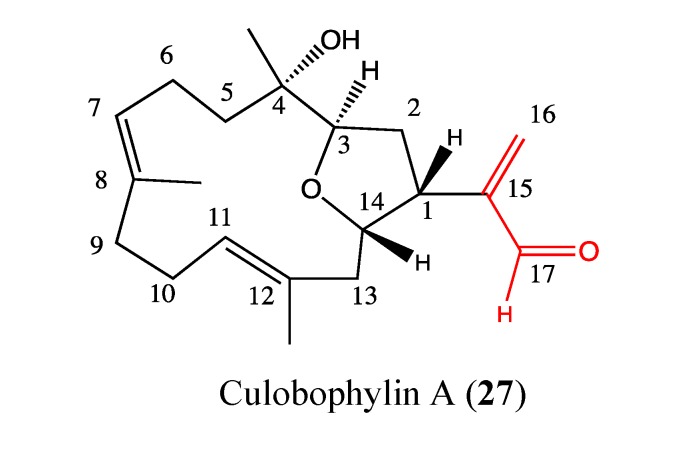
Chemical structure ofCulobophylin A.

Culobophylin A (**27**, Figure 20) is a novel cembranoid from diterpenoid families isolated from the cultured soft coral *Lobophytum crassum*. This compound exhibits high cytotoxicity against HL-60 and DLD-1 cells, with IC_50_ values of 3 and 4.6 μg/mL, respectively. No effect was observed on the expression of COX-2 and iNOS proteins in RAW264.7 macrophages implying a probable absence of anti-inflammatory activity [104]. This compound has unusual features in its chemical structure, which could be an asset for its pharmacological properties. The structure-function relationship studies show that the acetaldehyde moiety at C-15 is crucial for its cytotoxicity. Culobophylin A requires further investigation to elucidate its mechanism of action and to determine its possibilities for cancer therapy.

#### 2.9.3. Hippolide A

**Figure 21:**
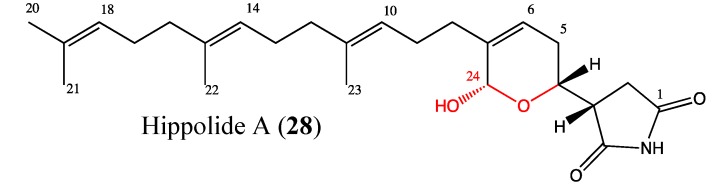
Chemical structure of Hippolide A.

Hippolide A (**28**, Figure 21) is an acyclic manoalide derivative that was isolated from the marine sponge *Hippospongia lachne*. Acyclic manoalides are sesterterpenoids that possess a terminal geranyl group instead of the cyclohexene ring commonly found in manoalide derivatives [105]. Hippolide is cytotoxic against a panel of cancer cell lines, including A549 (IC_50_ of 52.2 nM), HeLa (IC_50_ of 48 nM) and HCT-116 (IC_50_ of 9.78 μM). The cytotoxicity of this compound is linked to the C-24 acetal group in its chemical structure [106]. This novel compound could be considered for further biological investigations to determine its possible uses in cancer therapy.

#### 2.9.4. Irciformonin Analogs

**Figure 22:**
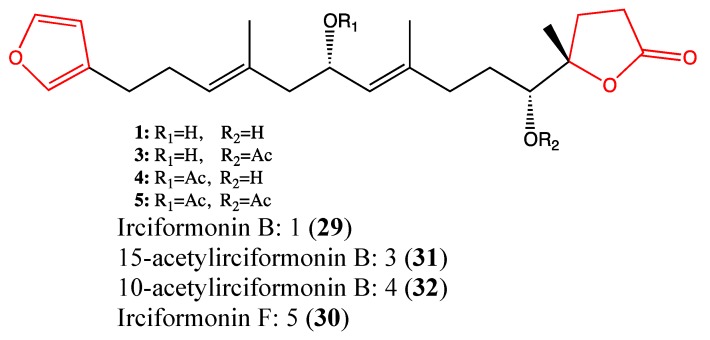
Chemical structure of Irciformonin analogs.

Irciformonin analogs are triterpenoid-derived metabolites that were isolated from the marine sponge *Ircinia* sp [107]. Their chemical structures are similar, and they exhibit high cytotoxicity against a panel of cancer cell lines, including K562, DLD-1, HepG2 and Hep3B cells. Four analogs, namely irciformonin B (**29**, Figure 22), F (**30**), 15-acetylirciformonin B (**31**) and 10-acetylirciformonin B (**32**) were observed to be cytotoxic toward K562, DLD-1, HepG2 and Hep3B cells, with IC_50_ values ranging from 0.03 to 10.2 μM [108]. Regarding the chemical structure of these compounds, it appears that the furan moiety is highly implicated in their cytotoxic effects. Recently, Su and co-workers reported that the DNA damage and apoptotic effects of 10-acetylirciformonin B in leukemia HL-60 cells occurs through the activation of caspases 3, 8 and 9 as well as through PARP cleavage [109]. Due to their high toxicity, these terpenoids require further investigation.

#### 2.9.5. Diterpene Isonitriles 1, 2, 3 and 4

**Figure 23:**
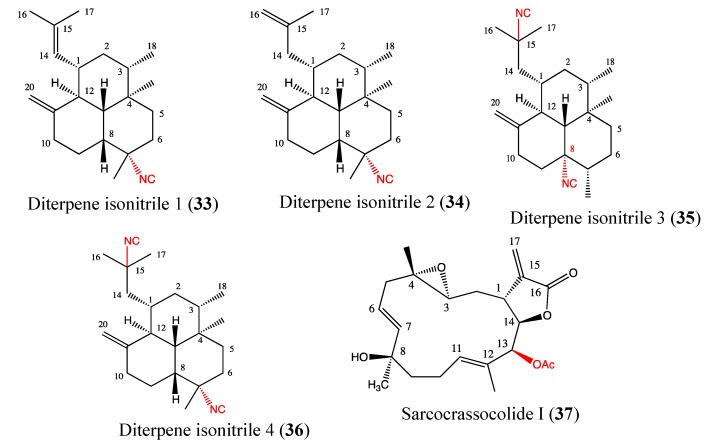
Chemical structure of Diterpene isonitriles and Sarcocrassocolide I.

Diterpene isonitriles are one of the most potent antiplasmodial compound groups characterized by an amphilectane skeleton [110]. It was known that several antimalarial molecules exert important anticancer properties [68]. The diterpene isonitriles 1–4 (**33**, **34**, **35**, **36**, Figure 23) were isolated from the Caribbean sponge *Pseudoaxinella flava*, and their chemical structures differ only in the number and position of the isonitrile groups and double bonds. Each of these analogs exhibited significant inhibition of growth in human prostate cancer cells (PC3), with the IC_50_ values ranging from 1 to 7 μM [111]. The most active analog is diterpene isonitrile 3, with IC_50_ of 1 ± 1 μM. This compound displays an important antiproliferative effect against LoVo colon cancer cells (IC_50_ of 3 ± 1 μM) and SKMEL-28 melanoma cells (IC_50_ of 6 ± 1 μM), whereas compound 4 is cytotoxic to U373 glioblastoma (IC_50_ of 10 ± 1 μM), Hs683 oligodendroglioma (IC_50_ of 4 ± 1 μM), and LoVo cells (IC_50_ of 3 ± 1 μM) [111]. Based on these results, the cytotoxic effects appear to be linked to the number and position of the isonitrile groups in the chemical skeleton of the compounds. The preliminary data suggest that diterpene isonitriles 1–4 are interesting analogs to be investigated in anticancer drug development.

#### 2.9.6. Sarcocrassocolide I

Sarcocrassocolide I (**37**, Figure 23) is a novel diterpenoid that was isolated from a soft coral *Sarcophyton crassocaule* and reported by Lin and coworkers in 2011 [112]. Its chemical structure is similar to that of culobophylin. This compound showed high cytotoxicity toward Daoy, Hep-2, MCF-7 and WiDr cells, with IC_50_ values ranging between 5 and 8 μM. Moreover, this compound displays significant anti-inflammatory activities through the inhibition of iNOS protein expression and a reduction of COX-2 protein levels [112]. The structure-function relationship studies showed that the acetoxy group at C13 is crucial for its cytotoxicity. 

#### 2.9.7. Sarcophine Analogs No 10 and No 13

**Figure 24:**
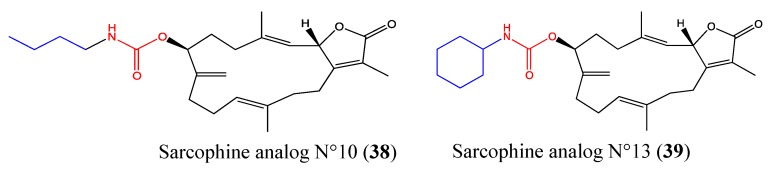
Chemical structure of Sarcophine analogs.

Sarcophine is a cembranoid diterpene that was isolated from the Red Sea soft coral *Sarcophyton glaucum*. The study of its structure-function relationship led to its semisynthetic analogs, which display an interesting anti-migratory property. Sarcophine analog No 10 (**38**, Figure 24) was found to possess significant anti-migratory activity against highly metastatic MDA-MB-231 breast cancer cells, with an IC_50_ value of 4.83 μM, whereas the analog No 13 (**39**, Figure 24) exerted its anti-migratory effect against PC-3 prostate cancer cells with an IC_50_ value of 15.53 μM. Interestingly, 5 and 30 μM doses of No 10 and No 13, respectively, induced anti-migratory effects comparable to those of a 200 μM dose of the reference drug 4-hydroxyphenylmethylene hydantoin (PMH) without affecting cell shape or viability [113]. These two compounds may be relevant in combination therapies and require further investigation.

#### 2.9.8. Siphonaxanthin

**Figure 25:**
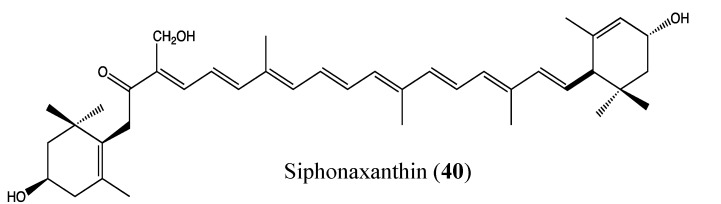
Chemical structure of Siphonaxanthin.

Siphonaxanthin (**40**, Figure 25) is a marine keto-carotenoid that was isolated from the siphonaceous green algae *Codium fragile*. It exhibits significant cytotoxicity against HL-60 human leukemia cells, with IC_50_ values between 5 and 10 μM. It also induces apoptosis in HL-60 cells through caspase-3 activation, which has been associated with the enhancement of GADD45α and DR5 expression levels as well as the suppression of Bcl-2 expression [114]. GADD45α is an important apoptosis regulator that induces cell cycle arrest, and DR5 is a death receptor. This compound was previously reported to possess anti-angiogenic properties in human umbilical vein endothelial (HUVEC) cells at a dose of 10 μM [115]. Recently, cancer chemoprevention and chemotherapy using nutraceuticals have been considered to be interesting approaches to reduce cancer incidence; thus, the carotenoid like siphonaxanthin and astaxanthin could be potential chemopreventive or chemotherapeutic agents.

#### 2.9.9. Smenospongine

**Figure 26:**
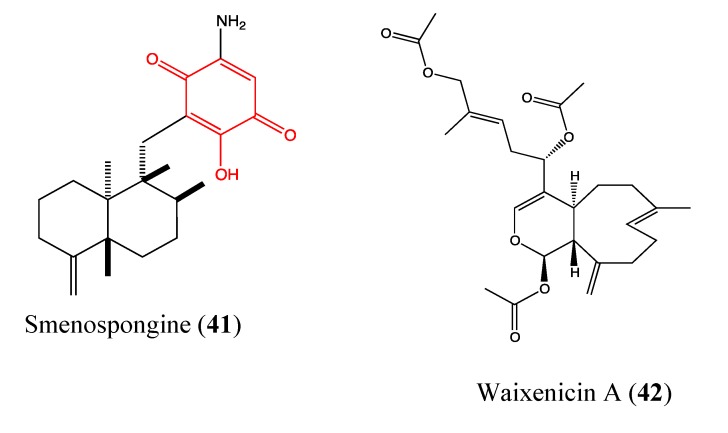
Chemical structure of Smenospongine and Waixenicin.

Smenospongine (**41**, Figure 26) is a sesquiterpene aminoquinone that was isolated from the Indonesian marine sponge *Dactylospongia elegans*. Previous studies showed that this sesquiterpene induces cell cycle arrest at G1 phase in chronic myelogenous leukemia (CML) cells as well as apoptosis in acute myelogenous leukemia (AML) and lymphocytic leukemia cells [116,117]. In 2011, Kong and co-workers reported that smenospongine exhibits significant inhibition of proliferation in human umbilical vein endothelial (HUVEC) cells in a dose-dependent manner, with an IC_50_ value of 4.9 μM [118]. It blocks both HUVEC migration and inhibits tube formation in a dose-dependent manner, which suggests that this sesquiterpene could be a promising antiangiogenic agent. Additionally, smenospongine is highly cytotoxic to both colorectal and lung cancer cells [118]. The presence of quinone and phenol hydroxyl moieties could explain the observed properties of this compound. 

#### 2.9.10. Waixenicin A

Waixenicin A (**42**, Figure 26) is a diterpenoid that was isolated from marine soft coral *Sarcothelia edmondsoni* [119]. It is found to significantly inhibit growth and proliferation of human Jurkat T-cells and rat basophilic leukemia (RBL1) cells at 300 nM with low cytotoxicity. Zierler and co-workers [120] demonstrated that waixenicin A is a potent and relatively specific inhibitor of transient receptor potential melastatin 7 (TRPM7) channels. TRPM7 is overexpressed in a variety of human cancer cells such as gastric adenocarcinoma, breast cancer and human head and neck carcinoma cells [121,122,123,124]. This protein contains an ion channel and a functional α-kinase domain, both of which are implicated in Mg^2+^ homeostasis [125,126,127,128]. It is known that Mg^2+^ is involved in cell proliferation, and cancerous cell growth represents the most detrimental effect of deregulated proliferation [129,130,131,132]. Moreover, TRPM7 regulates the migration of human nasopharyngeal carcinoma cells, suggesting its implication in metastasis [133]; thus, TRPM7 inhibitors could be beneficial in cancer treatment. Waixenicin A significantly inhibits both overexpressed and native TRPM7 channels in human embryonic kidney (HEK293) cells at micromolar levels in a dose-dependent manner. Its effect is potentiated by intracellular free magnesium (Mg^2+^). The inhibitory effect of waixenicin A was considered to be relatively specific to TRPM7 [120], justifying the high interest of this compound and its analogs in cancer treatment.

## 3. Discussion

**Figure 27:**
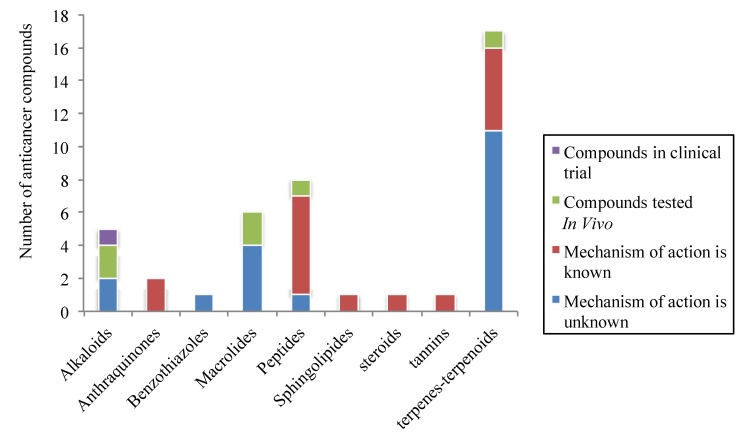
Anticancer compounds from marine origin regrouped per family and level of study.

According to our survey, the effort of many research groups to develop anticancer agents led to the publication of 42 molecules in 2011 which seem to be promising anticancer drugs. These compounds are regrouped into 9 classes of chemicals including alkaloids, anthraquinones, benzothiazoles, macrolides, peptides, sphingolipids, steroids, tannins, terpenes and terpenoids. The most representative are terpenes and terpenoids (40.48%) following by peptides (19.05%), macrolides 14.29%), and alkaloids (11.9%) (Figure 27). Among them, 50% are described for the first time as anticancer agents. The majority of these compounds are chemotherapeutic agents (92.7%) and only 7.3% are chemopreventives, which are known as nutraceutics available in fruits and vegetables. Many studies demonstrated the involvement of these nutraceuticals in cancer prevention or treatment [134,135,136]. The biological mechanisms involved in the anticancer properties of the investigated compounds are mainly cell cycle arrest through tubulin inhibitory effect; apoptosis through caspases 3, 8, 7, 9 activation, MMP depolarization, bib truncation, Bcl-xL, Bax and PARP cleavages, cytochrome c release, Bcl-2 and Akt down-regulation; anti-migratory effect through specific inhibitory effect against TRPM7 channels, anti-angiogenic property by inhibition of VEGF-A secretion; anti-inflammatory effect through the inhibition of COX-2 and iNOS expression. Surprisingly, the nuclear factor kappa-B (NF-κB) and the multi-drug resistant protein (P-gp), which are two important pharmacological targets seem to be less impacted by the investigated compounds. Indeed, only 4.76% and 2.38% of the 42 compounds inhibit NF-κB pathway and the expression of P-gp, respectively. Regarding the level of study of these compounds, only 2.38% are in clinical trial; 14.29% are tested *in vivo*. Among the 83.34% tested *in vitro*, the biological mechanism of action remains unknown for more than half of them (45.24%) (Figure 27). Beside the financial raisons, which could justify that situation, it is worth noting the fact that many compounds that showed interesting activities *in vitro* lose these properties when tested *in vivo*. This can be justified by the interactions between the compound and tested organism through many parameters including adsorption, distribution, metabolism and excretion [137]. Accordingly the physico-chemical properties like solubility and permeability of these compounds are mainly involved in their ability to modify the functional pathways *in vivo*. According to Lipinski *et al*., 4 parameters are globally associated with solubility and permeability, namely molecular weight, Log P, the number of H-bond donors and the number of H-bond acceptors, leading to the “rule of 5”. It states that poor absorption or permeability can be observed in compounds that contains more than 5 H-bond donors and 10 H-bond acceptors, with molecular weight over than 500 and Log P over than 5. In general, if two parameters are out of this range, the concerned compound is more likely to lose its pharmacological properties when tested *in vivo* [138]. Among the 42 promising marine anticancer discussed in this review, 6 of them (14.28%) are out of the Lipinski rule of 5 considering only the number of H-bond donors or acceptors and the molecular weight. These compounds are chromomycin SA2, lobophorin C and D, jasplakinolide, (+)-spongiostatin1 and dieckol. The risk of therapeutic failure seems to be high for these compounds although some compounds do not necessarily respect the rule of 5. For these 6 compounds, the structure-activity relationship studies may lead to some chemical modifications in order to reduce the molecular weight and the number of H-bond donors or acceptors without affecting the pharmacological properties.

## 4. Conclusion

This review provides an overview of 42 marine compounds that could be promising anticancer drugs. These compounds are characterized by their complex and/or unique chemical structure as well as their display of a large variety of biological activities, including antiproliferative, cytotoxic and antimetastatic properties. This diversity of structure and function highlights the great richness of marine organisms as an important source of new anticancer drugs. Thus far, structure-function studies appear to be a good approach for finding analogs with a high positive risk-benefit ratio. Each year, several new anticancer molecules were either isolated or synthetized; however, the majority of these molecules remain in preclinical investigation stages while patients are waiting for an alternative treatment option without the disadvantages or severe side effects of chemotherapy. 

## Abbreviations

### Cell Line Abbreviation

A549: Lung carcinoma cell line
A2780 and OVCAR-3: Ovarian cancer cells
ACHN: Human kidney adenocarcinoma cell
AGS: Human stomach adenocarcinoma cell line
CEM: leukemia cell
DLD1: Human colorectal carcinoma
Daoy: medulloblastoma cells
H1395 and H2122: Lung cancer cell
HCC44 and HCC366: non-small lung cancer cells
HCT116: Human colon carcinoma cell line
HEp-2: Human epithelial cells
Hep3B: Human hepatoma cells
HepG2: Human hepatocellular liver carcinoma cell line
Hel: Human erythroleukemia cells
HL-60: Human promyelocytic leukemia cell line
HT29: Human colon adenocarcinoma grade II cell line
HT1080: Fibrosarcoma cell line
IOSE-144: Non-tumorigenic human Ovarian surface epithelial cells
JB6 Cl 41: Mouse epidermal cell line
Jurkat: Human T-cell leukemia cell line
K562: Human chronic myeloid leukemia cell line
KB: human nasopharynx carcinoma cell line
MDR-KBv200: Multidrug resistant form of KB
LoVo: Colon cancer cell line
MCF-7: Breast adenocarcinoma cell line
MDA-MB231: Mammary carcinoma cell line
MiaPaCa2: Human pancreatic carcinoma cell line
PC-3: Prostate cancer cell line
RAW264.7: Mouse macrophage cell line
RPMI8226: Human multiple myeloma cell line
S180: Murine sarcoma cell line
U2OS: Human osteosarcoma cell line
U937-GTB: Histiocytic lymphoma cell line
WiDr: Colorectal adenocarcinoma cell line

### Others

1403P-3: Adriamycin analogue
AglRhz: Aglycon of Rhizochalin
AMPK: 5' adenosine monophosphate-activated protein kinase
AP-1: Activator protein 1
Bcl-2: B-cell lymphoma 2
Bax: Bcl-2-associated X protein
BF65 and BF78: Hemiasterlin derivatives
BRCA1: Breast cancer 1
CDK4: Cyclin-dependent kinase 4
FBA-TPQ: Makaluvamine analog
GADD45α: Growth arrest and DNA damage gene
IGF: Insulin-like growth factor
iNOS: Inducible nitric oxide synthase
MMP-9: Matrix metalloproteinase
MDR: Multidrug resistance
MESP: Methyl spongoate
NF-κB: Nuclear factor-kappa B
PARP: Poly (ADP-ribose) polymerase
P-gp: P-glycoprotein
PI3 kinase/Akt: Phosphoinositol-3-kinase
RAD51 and RAD54: DNA repair protein
ROS: Reactive oxygen species
S4-1e: Apratoxin analog
STAT3: Signal transducer and activator of transcription 3
TNFα: Tumor necrosis factor α
TRPM7: Transient receptor potential melastatin 7 channels
VEGF: Vascular endothelial growth factor
XIAP: X-linked inhibitor of apoptosis protein
